# Improving treatment outcome assessment in a mouse tuberculosis model

**DOI:** 10.1038/s41598-018-24067-x

**Published:** 2018-04-09

**Authors:** Bas C. Mourik, Robin J. Svensson, Gerjo J. de Knegt, Hannelore I. Bax, Annelies Verbon, Ulrika S. H. Simonsson, Jurriaan E. M. de Steenwinkel

**Affiliations:** 1 Department of Medical Microbiology & Infectious Diseases, Erasmus University Medical Center, Rotterdam, The Netherlands; 2 Department of Pharmaceutical Biosciences, Uppsala University, Uppsala, Sweden; 3 Department of Internal Medicine, Section of Infectious Diseases, Erasmus University Medical Center, Rotterdam, The Netherlands

## Abstract

Preclinical treatment outcome evaluation of tuberculosis (TB) occurs primarily in mice. Current designs compare relapse rates of different regimens at selected time points, but lack information about the correlation between treatment length and treatment outcome, which is required to efficiently estimate a regimens’ treatment-shortening potential. Therefore we developed a new approach. BALB/c mice were infected with a *Mycobacterium tuberculosis* Beijing genotype strain and were treated with rifapentine-pyrazinamide-isoniazid-ethambutol (R_p_ZHE), rifampicin-pyrazinamide-moxifloxacin-ethambutol (RZME) or rifampicin-pyrazinamide-moxifloxacin-isoniazid (RZMH). Treatment outcome was assessed in n = 3 mice after 9 different treatment lengths between 2–6 months. Next, we created a mathematical model that best fitted the observational data and used this for inter-regimen comparison. The observed data were best described by a sigmoidal E_max_ model in favor over linear or conventional E_max_ models. Estimating regimen-specific parameters showed significantly higher curative potentials for RZME and R_p_ZHE compared to RZMH. In conclusion, we provide a new design for treatment outcome evaluation in a mouse TB model, which (i) provides accurate tools for assessment of the relationship between treatment length and predicted cure, (ii) allows for efficient comparison between regimens and (iii) adheres to the reduction and refinement principles of laboratory animal use.

## Introduction

Tuberculosis (TB) claimed 1.7 million lives in 2016, which is more than any other infectious disease caused by a single pathogen^1^. The global treatment success rate for drug-susceptible TB is 83%, which falls short of the ≥90% target rate set by the WHO^1^. Moreover, treatment success rates against multi-drug resistant (MDR; 52%) and extensively drug resistant (XDR; 28%) TB are markedly lower^1^. These rates emphasize the need for more effective anti-TB drug regimens that can improve treatment success. In addition, new anti-TB regimens should allow for shortening of the current 6-months treatment length to increase compliance and minimize further drug resistance development.

Recently, a large clinical Phase III trial failed to reduce anti-TB treatment length from six to four months by substituting conventional anti-TB drugs with moxifloxacin^2^. This trial was conducted based on promising results from clinical Phase IIa/b trials and preclinical experiments in mouse TB models^3–5^. Overall, this has led to the conclusion that early surrogates for treatment efficacy assessments as measured in clinical phase IIa/b trials are unreliable predictors for cure in TB^6,7^. This has further inspired efforts to improve preclinical mouse TB models aimed at evaluating treatment outcomes^8^.

Preclinical evaluation of TB treatment outcome occurs primarily in mouse models^9^. The conventional design involves a two-step approach. During the first step, early treatment efficacy is measured by determining mycobacterial load reductions in the lungs of small groups of mice (n = 3–5) at preset time points until culture conversion is reached^10–15^. In the second step, relapse of infection is evaluated for regimens that resulted in culture negativity. This occurs by determining lung culture status three months after treatment has ended in larger groups of mice (n = 12–30), after 1–3 selected treatment lengths)^10–15^.

This conventional design seems to have several drawbacks. Most importantly, it allows for relapse rate comparison between regimens at selected time points, but does not provide an individual regimen’s correlation between treatment length and treatment outcome. This correlation is required to efficiently estimate a regimen’s treatment-shortening potential. The conventional design also has limited screening potential for regimens with unknown efficacy, as prior knowledge on when a regimen will reach culture-conversion is required before relapse can be evaluated. Lastly, recent clinical and preclinical observations suggest that early treatment efficacy assessment as measured in step one of the conventional design has limited predictive value for treatment outcome after a full course of anti-TB treatment^6,7,16^.

In the current study we propose an alternative design for treatment outcome assessment in our mouse TB model. We increase the number of treatment schedules assessing outcome three months after the end of treatment regardless of culture status at the end of treatment, but decrease the number of mice per treatment length (n = 3 instead of n = 12–30). This way of data collection allows for mathematical modeling of the observational data optimized for establishing a robust and informative link between treatment length and cure.

The mathematical modeling is based on conventional logistic regression, but is designed to be more informative. This approach differs from survival-, or time-to-event analysis, because the bacterial burden is determined after a fixed period of time after stop of treatment. Therefore the time of culture-conversion relative to stop of treatment is unknown.

*In silico* simulations of the mathematical model can be used to visualize and accurately quantify the association between treatment length and predicted treatment outcome for each regimen. Advantages include the possibility to compare the curative potential of different anti-TB regimens with each other over time instead of at selected time points only and simultaneously assess the treatment-shortening potential of each individual regimen.

## Material and Methods

### Mice, infection and mycobacterial strain

Specified pathogen-free female BALB/c mice, aged 13–15 weeks, were infected by intratracheal installation of 1.0–1.8 × 10^5^ drug-susceptible *Mycobacterium tuberculosis* Beijing VN 2002–1585 (BE-1585) under general anesthesia as described previously^16,17^. The mice were housed and experiments were conducted in the Erasmus MC animal biosafety level III facility.

### Ethical approval

All protocols were approved by the Erasmus MC animal ethics committee under DEC number 117-12-13 and EMC number 2887, and were in accordance with the rules laid down in the Dutch Animal Experimentation Act and the EU Animal Directive 201/63/EU.

### Treatment

Treatment consisted of either of three regimens: (i) rifapentine, pyrazinamide, isoniazid and ethambutol (R_p_ZHE), (ii) rifampicin, pyrazinamide, moxifloxacin and ethambutol (RZME) or (iii) rifampicin, pyrazinamide, moxifloxacin and isoniazid (RZMH). The first two months of each regimen consisted of treatment with all four drugs (intensive phase) followed by four months of treatment with rifapentine and isoniazid for the R_p_ZHE regimen, rifampicin and moxifloxacin for RZME and rifampicin, moxifloxacin and isoniazid for RZMH. All drugs were administered 5 days a week via oral gavage in their human pharmacokinetic equivalent dose: rifampicin: 10 mg/kg, rifapentine: 10 mg/kg, moxifloxacin: 200 mg/kg, isoniazid: 25 mg/kg, ethambutol: 100 mg/kg, pyrazinamide: 150 mg/kg^18,19^.

### Treatment outcome evaluation

Treatment was initiated 2 weeks after infection and was stopped between 2 and 6 months with intervals of 2 weeks (i.e. nine different treatment lengths per drug regimen). The protocol was designed to include three (n = 3) mice per treatment length. A sample size of n = 3 was found to be sufficient to detect a 50% difference in potency between different treatments and was expected to give reasonably high precision in model parameters, according to a statistical power calculation (described in Supplementary data file S1).

One ‘backup’ mouse was added per regimen to reduce the impact of unexpected animal loss. All mice were sacrificed 3 months post-treatment to determine mycobacterial loads in the lungs as described previously^16^.

### Statistical analysis

The statistical analysis involved the development of a logistic regression model based on the observational data. These data were treated as a binary outcome variable of either cure (defined as a negative solid culture 3 months post-treatment) or failure (defined as a positive solid culture 3 months post-treatment). The independent variable was treatment length. The data were analyzed using the non-linear regression software NONMEM (version 7.3) with simultaneous estimation of all model parameters^20^. Only if parameters were estimated close to a parameter boundary (as described below) they were fixed to the value of the respective boundary. NONMEM maximizes the likelihood of a model to fit the observational data. In NONMEM the model fit (defined as the likelihood of the model to describe the observational data) was assessed using the objective function value (OFV), which is equal to - 2 times the log value of the likelihood. In order to generate a model that best described (fitted) the data, the OFV between models was compared using the likelihood ratio test (LRT). To this aim, for each model comparison a reduced model and a full model were evaluated where the full model always included more model parameters than the reduced model. The null hypothesis was that the full model did not provide better fit than the reduced model. Testing was performed at the 5% significance level which corresponds to a drop in the OFV of at least 3.84 points with one degree of freedom. Data handling and graphical analysis were conducted in R (version 3.3.0)^21^.

The model development was divided into two parts; in the first part, an appropriate relation between probability of cure and treatment length was identified (regardless of drug regimen). In the second part we explored if this relation between probability of cure and treatment length was significantly different between the drug regimens.

The starting point for the first part of model development was a base model which assumed that the probability of cure was identical regardless of treatment lengths according to:

1 $${p}_{failure}=1-{p}_{cure}={p}_{base}$$

In this model, p_failure_ and p_cure_ are the predicted probabilities of failure and cure respectively and p_base_ is the base probability of failure. The p_base_ parameter was constrained to be between 0 and 1. First, this base model was compared to a model assuming linear increase in cure rate with treatment length according to:

2 $${p}_{failure}=1-{p}_{cure}={p}_{base}\times (1-Slope\times T)$$

In this model ‘Slope’ is the linear increase in probability of cure with treatment length (T). The ‘Slope’ parameter was constrained to be between 0 and 1 divided by the maximum treatment duration of 6 months. Secondly, an E_max_ model was tested according to:

3 $${p}_{failure}=1-{p}_{cure}={p}_{base}\times (1-\frac{{E}_{max}\times T}{{T}_{50}+T})$$

In this model ‘E_max_’ is the maximal achievable probability of cure and ‘T_50_’ is the treatment length at which half the E_max_ is seen. The E_max_ parameter was constrained to be between 0 and 1. Lastly, a sigmoidal E_max_ model was tested according to:

4 $${p}_{failure}=1-{p}_{cure}={p}_{base}\times (1-\frac{{E}_{max}\times {T}^{\gamma }}{{T}_{50}^{\gamma }+{T}^{\gamma }})$$

In this model ‘γ’ is a shape parameter controlling the steepness of the curve produced by the E_max_ equation.

In the second part of model development, we explored if the identified relation between cure and treatment length from the first part of model development was significantly different for the different drug regimens by comparing the model parameters of the different drug regimens (Slope, E_max_, T_50_ or γ, depending on the model). This was done in a step-wise approach, here exemplified for a sigmoidal E_max_ model, which includes the three model parameters E_max_, T_50_ and γ. Firstly, one model was fitted to explore if E_max_ for each regimen was significantly different from the other two regimens. This procedure was repeated for the T_50_ and γ parameters, thus resulting in nine different models. Secondly, the models that did not significantly improve the fit (OFV drop of less than 3.84 points) were not evaluated further. Of the remaining models that did result in an OFV drop of at least 3.84 points, the model with the lowest OFV was accepted. Thirdly, the accepted model with the greatest drop in OFV was combined with the remaining models that also improved the fit significantly (i.e. whose OFV drop was lesser than the accepted model but at least 3.84 points). If this combination improved the fit significantly, it was accepted as the new model. This whole three-step procedure was repeated until no significant improvement was seen anymore, which was defined as the final model.

In addition to assessment of OFV, model selection was guided by parameter uncertainty and visual predictive checks (VPC) generated using PsN (http://psn.sourceforge.net/ [cited 19-12-2016]) and Xpose (http://xpose.sourceforge.net/ [cited 19-12-2016]) using 1000 simulated datasets. The VPC is a visual diagnostic which shows how well data simulated from a model agree with the observed data.

### Simulations

The observational data using n = 3 animals can only theoretically generate cure rates of 0%, 33%, 67% or 100%. Therefore, we used the mathematical model to simulate treatment outcome from 1000 mice per time point to increase the resolution in the predicted cure rates (i.e. to allow cure rate to continuously range between 0–100%). Simulations were performed using Monte Carlo sampling from a random uniform distribution ranging from 1 to 0. This was also used to determine the model-predicted treatment length required for each regimen to achieve 85%, 90% or 95% cure, respectively.

### Data availability

All data generated or analysed during this study are included in this published article (and its Supplementary data files).

## Results

### Observed treatment outcome

**Figure 1:**
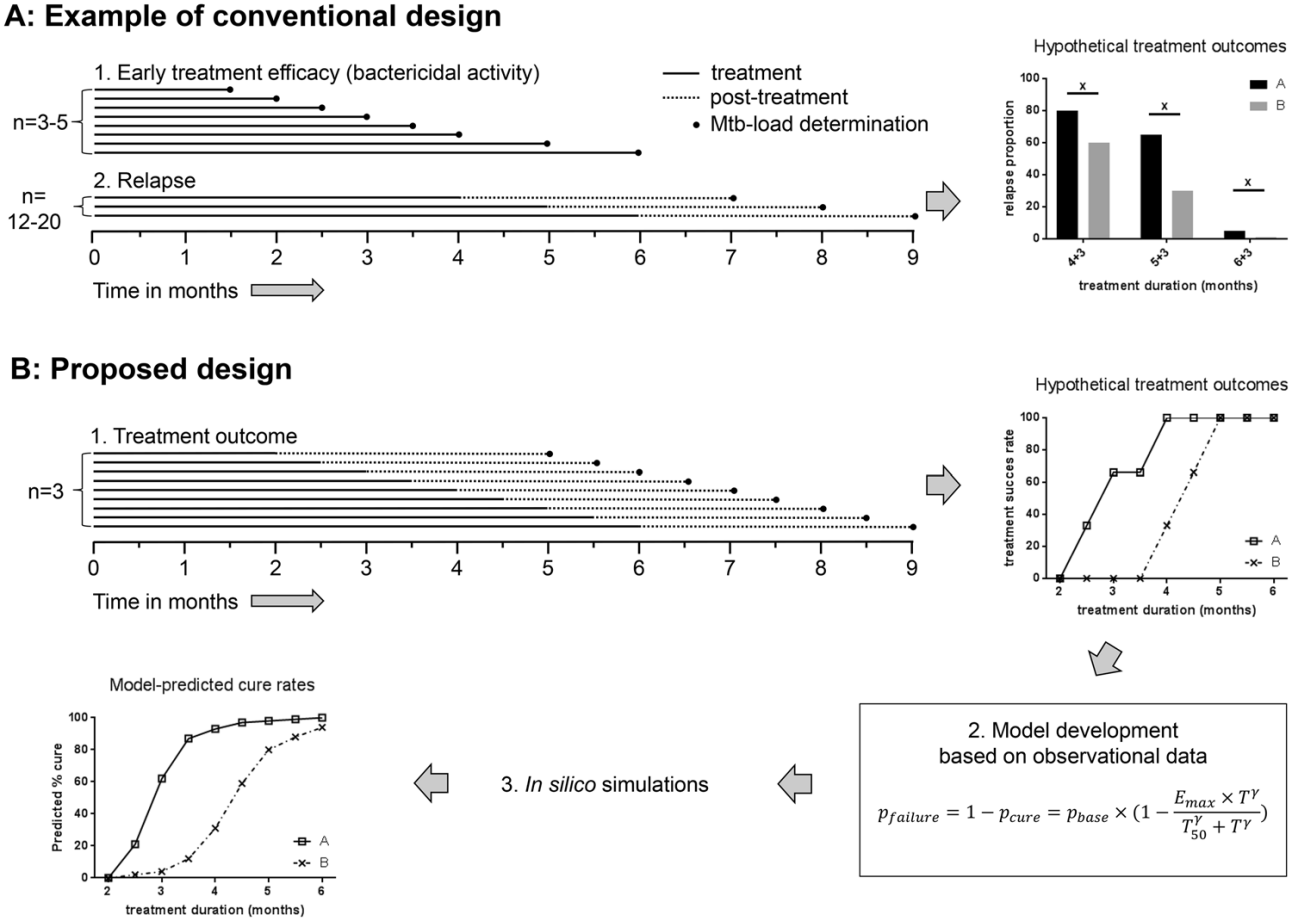
Schematic examples of the conventional design and the proposed design for treatment outcome evaluation in mouse TB models. (**A**) Shows an example of the conventional design in which bactericidal activity is determined by measuring reductions in Mtb-loads in the lungs until culture negativity is reached, followed by cross-sectional evaluation/comparison of relapse rates 3 months post-treatment (x in upper right figure). (**B**) Shows our proposed design in which treatment outcome is determined regardless of lung culture-status at stop of treatment. This allows for more informative mathematical modeling of the data and subsequent simulation of large numbers of mice to generate a high resolution correlation between treatment duration and treatment success.

A schematic overview of our method of data collection compared to the conventional design is found in Fig. 1. The observed proportions of cured animals for the different treatment lengths for the R_p_ZHE, RZME and RZMH regimens are shown in Table 1. R_p_ZHE started to show cure rates above 0% after 2.5 months of treatment and showed 100% cure after 4 months. RZME displayed similar kinetics and also showed complete cure rates from 4 months of treatment onwards. In contrast, RZMH only started to show cure rates above 0% after 4 months of treatment and did not reach complete cure even after 6 months of treatment.

**Table 1:** Observational data on cure.

Treatment length	R_p_ZHE	RZME	RZMH
2 months	0/3	0/3^a^	0/3
2.5 months	1/3	0/3	0/3
3 months	2/3	3/3	0/3
3.5 months	2/3	2/3	0/3
4 months	3/3	2/2^b^	1/3
4.5 months	2/2^c^	3/3	2/3
5 months	3/3	3/3	3/3
5.5 months	3/3	2/2^b^	3/3
6 months	3/3	3/3	3/4^d^

^a^0/3 = 0 of 3 mice was cured (culture-negative lungs 3 months post-treatment) after indicated treatment duration; ^b^Animal died of a non-tuberculosis cause prior to time point; ^c^The plates for colony counting were contaminated and no counting could be performed; ^d^The backup mouse included for the RZMH regimen was still alive at the 6 month time point. R = rifampicin, R_p_ = rifapentine, Z = pyrazinamide, M = moxifloxacin, H = isoniazid, E = Ethambutol.

### Model development

#### Part I: Relation between treatment length and probability of cure

The observational data from Table 1 were first converted into a dataset used for modeling (Supplementary data file S2). Compared to the base model which does not assume any relationship between cure and treatment length, a linear relationship between treatment length and cure gave a significant improvement in model fit compared to the base model (p < 0.001, OFV drop of 32.2 points). An E_max_ relationship between treatment length and cure did not improve model fit compared to a linear relationship (OFV increased with 15.9 points) and was rejected. However, a sigmoidal E_max_ relationship improved model fit significantly compared to a linear relationship between treatment length and probability of cure (p = 0.001, OFV drop of 13.3 points). Thus, the sigmoidal E_max_ model was identified as appropriate and was brought forward to the second part of model development.

Notably, the baseline probability (p_base_) in this sigmoidal E_max_ model was estimated very close to 1, which resulted in an unstable model (not possible to obtain any parameter uncertainty). Fixing p_base_ to 1 could correct for this without affecting the OFV.

#### Part II: inter-regimen differences

The generated sigmoidal E_max_ relationship in part 1 of model development assumed a similar relationship between treatment length and probability of cure for all three regimens tested. In order to detect if the selected relationship deviated significantly between the different regimens, we determined if implementing drug regimen-specific model parameters (including γ, E_max_ and T_50_) improved model fit and could detect significant differences between the different regimens.

Initially, the following models improved the model fit to the observational data significantly: Model 1: separate T_50_ for RZMH (p < 0.001, OFV drop of 18.6 points), Model 2: separate E_max_ for RZMH (p = 0.00298, OFV drop of 8.82 points), Model 3: separate γ for RZMH (p = 0.0408, OFV drop of 4.18 points) and Model 4: separate T_50_ for R_p_ZHE (p = 0.0377, OFV drop of 4.32 points). Model 1 (separate T_50_ for the RZMH regimen) had the lowest OFV and was therefore accepted. When combined with model 2–4, no significant improvements were observed. Therefore only model 1, which included simultaneous estimation of separate T_50_ parameters for RZMH only and for RZME and RpZHE, respectively was selected.

Notably, The E_max_ parameter for the sigmoidal E_max_ model with a separate T_50_ for the RZMH regimen was estimated very close to 1 which also resulted in an unstable model. Fixing E_max_ to 1 could correct for this and improved the model fit slightly (p = 0.827, OFV drop of 0.048 points).

Taken together, the final model included a sigmoidal E_max_ relationship where the probability of cure increased with treatment length. The E_max_ parameter had the value of 1 which implies that all included regimens can achieve 100% cure if the treatment length is sufficiently long. The baseline probability (p_base_) also had a value of 1 which implies that at very short or no treatment duration at all (i.e. T = 0) treatment failure will occur in all mice. Apart from p_base_ and E_max_, all parameters were simultaneously estimated.

Importantly, our finding that estimating a separate T_50_ for the RZMH regimen significantly improved our model fit indicates that RZMH has reduced curative potential compared to the other regimens. The final model parameters are shown in Table 2. The final model code is supplied in Supplementary data file S3.

**Table 2:** Final parameter estimates.

Parameter	Description	Parameter estimate	Standard error (%CV)^a^
p_base_	Baseline probability of no cure	1 FIX	—
E_max_	Maximum achievable probability of cure	1 FIX	—
T50_RpZHE/RZME_ (months)^b^	The treatment time at which half the E_max_ is reached for R_p_ZHE and RZME	2.87	5.4
T50_RZMH_ (months)^b^	The treatment time at which half the E_max_ is reached for RZMH	4.35	6.0
γ	Shape factor	9.82	23.0

R_p_ = rifapentine, Z = pyrazinamide, M = moxifloxacin, H = isoniazid, E = Ethambutol; CV coefficient of variance; ^a^The standard errors were calculated using the covariance step in NONMEM; ^b^T50 was significantly different between treatment arms (no statistically significant differences were found in other parameters).

### Model validation

**Figure 2:**
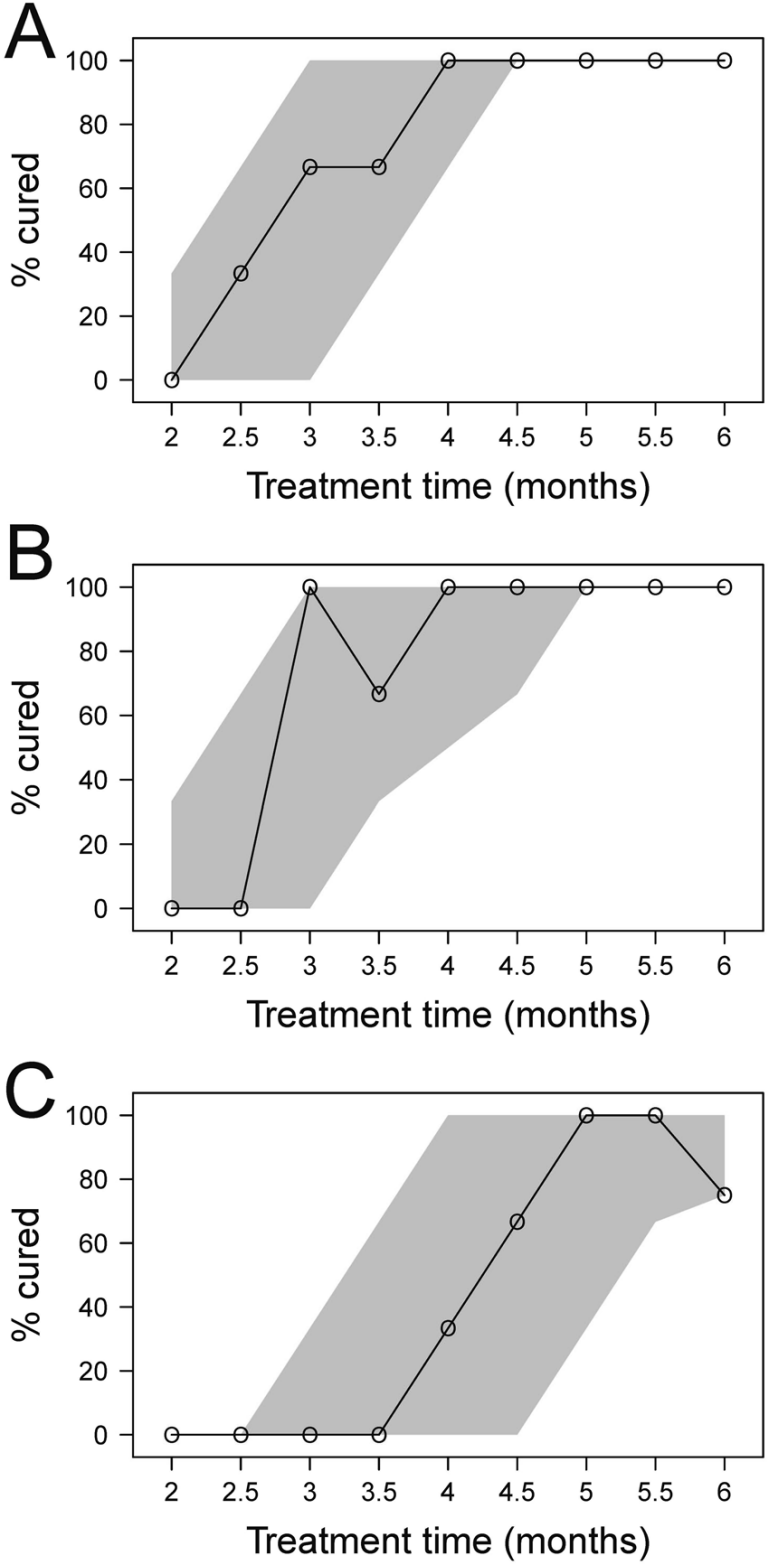
Visual predictive check (VPC) of the final model for each regimen. (**A**) rifapentine, pyrazinamide, isoniazid and ethambutol (R_p_ZHE), (**B**) rifampicin, pyrazinamide, moxifloxacin and ethambutol (RZME) and (**C**) rifampicin, pyrazinamide, moxifloxacin and isoniazid (RZMH). The open circles connected by the solid black lines are the observed probabilities of cure following different treatment lengths and the shaded areas are the 95% non-parametric confidence interval of the predicted cure rates following different treatment lengths.

To verify the model, a visual predictive check (VPC) was performed where the observational data and simulated data (presented as 95% confidence interval based on 1000 simulated datasets) were compared in the same plot (Fig. 2). As can be seen in the VPC, the observed proportions of cure fell within the 95% confidence of the simulated data. The confidence intervals may appear large at some time points which is due to the relatively low number of animals per time point and thus, given the data, the model can describe the observed data well.

#### Part I: Cure rate predictions based on model simulation

**Figure 3:**
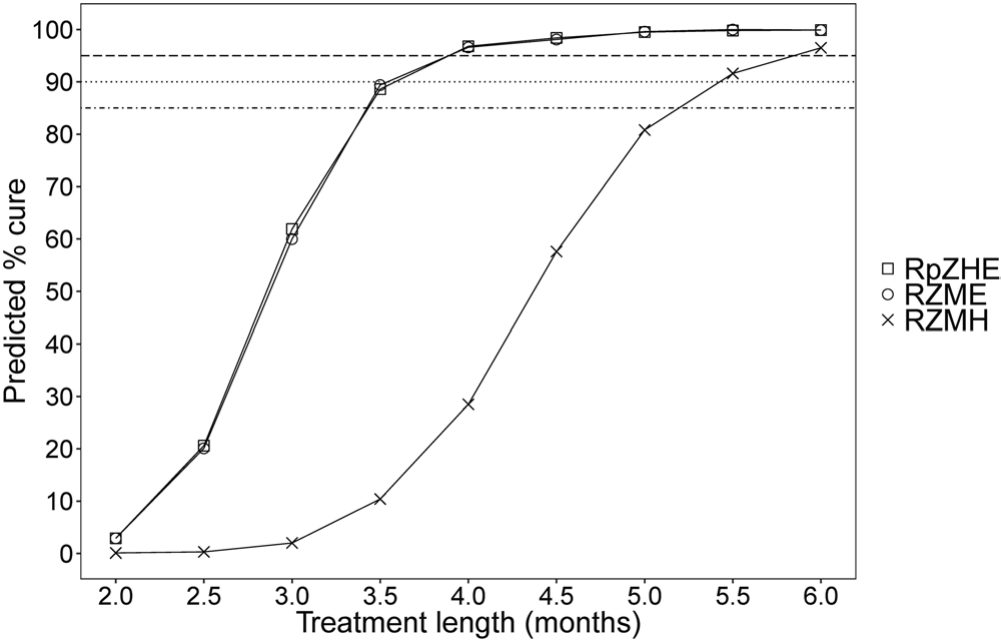
Model-predicted cure at different treatment lengths for each regimen. The black horizontal lines indicate 95% (dashed line), 90% (dotted line) and 85% (dashed-dotted line) cure rates. R = rifampicin, R_p_ = rifapentine, Z = pyrazinamide, M = moxifloxacin, H = isoniazid, E = Ethambutol.

Simulations of high numbers of mice (n = 1000 per arm) using the developed model enabled us to provide a high-resolution estimate of the predicted cure rates of each regimen for different treatment lengths as shown in Fig. 3. For R_p_ZHE and RZME this estimates that mice must be treated at least 3.5 months to reach 85% cure and 4 months to reach 90% or 95% cure. In contrast, mice must be treated with RZMH for at least 5.5 months to reach 85% or 90% cure and a full 6 months to reach 95% cure.

#### Part II: Model comparison to conventional relapse assessment other mouse TB models

Next, we aimed to evaluate if the predicted cure rates generated in our model were comparable to observational data obtained from other mouse TB models using pulmonary infection. These data are shown in Table 3^5,12,13,22,23^. A direct advantage of our model-based approach is the possibility to compare our predicted cure rates for any treatment length evaluated in other mouse TB models (Fig. 3 and Table 3).

**Table 3:** Comparison of our model-based predictions of cure rates with observational data.

	Regimen	% cured at:			Ref.
		3 months	4 months	5 months	
**Our model** BALB/c, Beijing, HDIT	2 R_p_ZHE/1,2,3 R_p_H^a^	62%	97%	100%	
	2 RZME/1,2,3 RM^a^	60%	97%	100%	
	2 RZMH/1,2,3 RMH^a^	2%	29%	81%	
**Model 1** BALB/c, H37Rv, HDA	2 R_p_ZM/1,2 R_p_M	100%			^22^
	3,4,5 RZM	75%	100%	100%	^5^
	2 RZM/1,2,3 RM	83%	100%	100%	^5^
	2 RZM/2,3 RM		95%	100%	^23^
**Model 2** BALB/c, H37Rv, LDA	2 RZME/1 RM	80%			^12^
	2 RZMH/1 RMH	27%			^12^
**Model 3** BALB/c, Erdman, HDA	2 RZM/2 RM		84%		^13^
**Model 4** BALB/c, Erdman, LDA	3 RZME	100%			^12^
	3 RZMH	93%			^12^
	2 RZM/1 RM, 2 RM	100%	95%		^13^
**Model 5** C3HeB/FeJ^b^, H37Rv, LDA	2 RZME/1 RM	80%			^12^
	2 RZMH/1 RMH	80%			^12^
**Model 6** C3HeB/FeJ^b^, Erdman, LDA	3 RZME	40%			^12^
	3 RZMH	0%			^12^

^a^Predicted cure for 3,4 and 5 months of treatment is shown as estimated in Fig. 3, ^b^C3HeB/FeJ mice can develop cavitating lesions that more closely resemble human disease, Abbreviations for route of infection: HDIT = high dose intratracheally, HDA = high dose aerosol, LDA = low dose aerosol, R = rifampicin, R_p_ = rifapentine, Z = pyrazinamide, M = moxifloxacin, H = isoniazid, E = Ethambutol.

Our model predicted a cure rate of 60% for RZME after three months of treatment. This was lower than the observed cure rates from three other mouse TB models, which were 80–100% (Table 3: models 2, 4, 5), but higher than the observed cure rate of 40% in model 6^12^. After four months of treatment no data on RZME was available in other mouse TB models and we could only compare our data to the RZM regimen. A 97% predicted cure rate for RZME in our model showed similar cure rates as observed for RZM in models 1 and 4 and a 13% higher cure rate than RZM in model 3 (Table 3). After five months of treatment, results were similar compared to one other mouse TB model, which also showed 100% cure.

For RZMH, our predicted cure rate of 2% after three months of treatment was lower compared to the observed cure rates of 27%, 93% and 80% in models 2, 4 and 5, respectively, but higher than the 0% cure observed in model 6 (Table 3). After four months, RZMH in our model could only be compared to RZM in other mouse TB models. Our predicted cure rate of 29% for RZMH at this point was markedly lower than the cure rates observed for RZM of 100–95%, 84% and 95% in models 1, 3 and 4 respectively. In this regard it is of note to mention that after three months of treatment RZMH also showed inferior results compared to RZM in mouse TB models 1 and 4 and inferior results compared to RZME in mouse TB models 2, 4 and 6 (Table 3).

Taken together, our finding that RZMH has significantly lower curative potential compared to RZME is reflected in trends observed in other mouse TB models. Moreover, the discrepancy between our predicted cure rates for RZMH compared to RZM in other mouse TB models, suggests a negative effect of H on the efficacy of RZM in mouse TB models.

## Discussion

In this study we demonstrated that a model-based analysis of observational *in vivo* data on TB treatment outcomes can be used to generate a high resolution association between treatment length and probability of cure. The developed model could detect statistically significant differences in the curative potentials of R_p_ZHE and RZME compared to RZMH, which could not have been identified based on the observational data alone. Validation of our model against other mouse TB models supported a negative effect of isoniazid on the efficacy of RZM in mouse studies.

In our model RZMH showed significantly reduced curative potential compared to RZME. Interestingly, a similar trend was observed in other mouse TB models where RZMH consistently showed a trend towards inferior results compared to RZME and/or RZM^12^. One explanation for this phenomenon might be a species-dependent, antagonistic effect of isoniazid on the therapeutic efficacy of rifampicin. Rifampicin is more essential for cure than isoniazid in mice^16^. It has been demonstrated that concomitant administration of isoniazid negatively affects the pharmacokinetics of rifampicin by lowering the highest observed plasma concentration (C_max_) and area under the plasma concentration-time curve (AUC)^24^. However, pharmacokinetics is an unlikely cause in our model as isoniazid co-administration previously did not affect rifampicin C_max_ and AUC compared to rifampicin monotherapy^25^. Also in patients no clinically significant pharmacokinetic interactions between isoniazid and rifampicin have been reported^26^. Nevertheless, addition of isoniazid (H) to the combination of rifampicin and pyrazinamide (RZ) significantly reduced bactericidal activity and cure in other mouse TB models^24,27,28^. In addition, an earlier study in our mouse TB model showed that RZ-treated mice had higher cure rates than mice treated with RH or RHZ after a six-months treatment course (95% vs. 87% and 80%, respectively)^16^. This previous comparison between RZ, RH and RHZ using the conventional design as shown in Fig. 1 did not yield significant differences, but the observed inferiority of RZMH compared to RZME in the current study supports earlier observations of an antagonistic effect of isoniazid on the therapeutic efficacy of rifampicin in mice.

Advantages of the combination of animal research and mathematical modeling are the ability to detect significant differences in the curative potential of different regimens, and the ability to compare our data with other studies that evaluated treatment outcome after any given treatment length as demonstrated in Table 3. In addition, animal research experiments should always strive towards the 3R-principles of replacement, reduction and refinement^29^. Our method adheres to the reduction and refinement principles. Firstly, the implementation of mathematical modeling and simulations can be considered a refinement as it enabled us to detect significant differences between regimens and allowed efficient comparison with other mouse TB models, which could not be derived from our observational data alone. Secondly, our approach enables assessment of treatment outcome without requiring early treatment efficacy data. This reduces the total number of mice required (Fig. 1).

Early treatment efficacy as measured through bactericidal activity might be of limited predictive value for treatment outcome in TB^16^. However, it remains an important screening tool in the setting of early drug discovery. The similar principle of observational data and mathematical modeling can be applied to bactericidal activity experiments as well using ‘culture negativity’ as outcome parameter in order to improve data interpretation.

One initial concern with the proposed design was that with only n = 3 mice per time point, the treatment outcome in a single mouse on a crucial time point might have a disproportional impact, e.g. if in the RZMH group 4/4 mice would be cured after 6 months or if only 2/3 mice would be cured in the RZME group after 6 months (Table 1). However, sensitivity analysis of such scenarios did not alter the conclusions based on the model (results not shown). This can be explained by the notion that the fit of a model involves all mice evaluated at all time points and thus reduces the impact of potential outliers at a single time point.

A common method to analyze binary data is standard logistic regression but in this work we applied a new alternative to standard logistic regression. The main advantage with our new method is that it is more widely applicable than standard logistic regression. Observational data may not always behave similar to a logistic curve and in such situation our new method will outperform logistic regression. Additionally our new method can detect differences in the maximum probability of cure which standard logistic regression cannot provide. Furthermore, if different mouse models are compared, the treatment failure rate at no treatment may be different (i.e. different p_base_ between mouse models) which is another example of a scenario that can be handled using our approach but not using conventional logistic regression.

A potential improvement of our model in its current form might be evaluation of the (re)growth curve of *M. tuberculosis* during treatment failure. In the current design, the data were analyzed as a binary outcome because cure or failure was based on the absence or presence of mycobacteria in the lungs at a single time point three months after stop of treatment. If mycobacterial loads were measured at multiple time points after stop of treatment, e.g. after one, two and three months, as opposed to only three months, a time-to-event approach could have been used to analyze the data. A time-to-event analysis is considered more informative than analyzing the data as a binary outcome because it can provide information on the time course of cure or relapsing treatment failure. This could allow for better estimation of treatment success rates, but would also require substantially more mice.

In conclusion, we provide a new design for treatment outcome evaluation in our mouse TB model, which (i) provides accurate tools for assessment of the relationship between treatment length and predicted cure, (ii) allows for efficient comparison between regimens, (iii) can be readily compared to other studies and (iv) adheres to the reduction and refinement principles of laboratory animal use.

## Electronic supplementary material

Supplementary data
