# Supplementary material for: Improving treatment outcome assessment in a mouse tuberculosis model
Source: Sci Rep. 2018 Apr 9;8:5714. doi: 10.1038/s41598-018-24067-x (PMC5890284; doi:10.1038/s41598-018-24067-x)
Supplement: Supplementary file 1 — Supplementary data [file 41598_2018_24067_MOESM1_ESM.docx]

**Improving treatment outcome assessment in a mouse tuberculosis model**

Bas C. Mourik^1^, Robin J. Svensson^2^, Gerjo J. de Knegt^1^, Hannelore I. Bax^3^, Annelies Verbon^3^, Ulrika S.H. Simonsson^2^, Jurriaan E.M. de Steenwinkel^1^

^1^ Department of Medical Microbiology & Infectious Diseases, Erasmus University Medical Center, Rotterdam, the Netherlands

^2^ Department of Pharmaceutical Biosciences, Uppsala University, Uppsala, Sweden

^3^ Department of Internal Medicine, Section of Infectious Diseases, Erasmus University Medical Center, Rotterdam, the Netherlands

**SUPPLEMENTARY DATA**

**Supplementary data file S1, Statistical power analysis**

**Aim**

To determine the appropriate sample size in order to detect a statistically significant difference in potency between different treatment regimens.

**Methods**

The statistical power to detect a statistically significant difference, at the 5% significance level, in potency of 50% between different treatments were conducted at different sample sizes (n=2-5 animals per time point). Included time points were 2 to 6 months with 0.5 month intervals (9 time-points in total). The power calculations assumed a sigmoidal E_max_ model with p_base_ of 1, E_max_ of 1, γ of 10 and a T_50_ of 2 months for treatment A (T_50_,A) and T_50_ of 4 months for treatment B (T_50_,B). One thousand simulations were performed at each sample size. The simulated data were re-estimated with one true model which included a difference in T_50_ between arms and an alternative model with no difference in T_50_. The proportion of re-estimated models where the true model (including difference between treatments) was significantly better than the alternative model (without difference between treatments) was the power at each sample size. In addition, the precision in the estimated parameters for the true model were also summarized. The estimated parameters were γ, T_50_,A and T_50_,B. E_max_ and p_base_ were both fixed to 1.

**Results**

The power to detect a significant difference in potency was: 87.9, 100, 100 and 100% for sample size of 2, 3, 4 and 5, mice per time point, respectively. This indicates that a sample size of 3 or more mice is sufficient to achieve 100% power to detect a statistically significant difference in potency between different treatments. The parameter precisions for n=3 expressed as relative standard error were 13.0, 0.8 and 0.2 % for γ, T_50_,A and T_50_,B, respectively which indicates that the expected parameter precision is reasonably high at n=3.

**Conclusion**

A sample size of n=3 mice per time point is sufficient to detect a 50% difference in potency between different treatments and also gave reasonably high precision in model parameters (i.e. low parameter uncertainty).

| **Supplementary data file S2, Modeling Dataset** | | | |
| --- | --- | --- | --- |
| **ID** | **COMB**  **(1: R_p_ZHE, 2:RZME, 3:RZMH)** | **CP**  **(CULTURE POSITIVE)** | **TIME (MONTHS)** |
| 1 | 1 | 3 | 2 |
| 2 | 1 | 3 | 2 |
| 3 | 1 | 3 | 2 |
| 4 | 1 | 0 | 2,5 |
| 5 | 1 | 3 | 2,5 |
| 6 | 1 | 3 | 2,5 |
| 7 | 1 | 0 | 3 |
| 8 | 1 | 0 | 3 |
| 9 | 1 | 3 | 3 |
| 10 | 1 | 3 | 3,5 |
| 11 | 1 | 0 | 3,5 |
| 12 | 1 | 0 | 3,5 |
| 13 | 1 | 0 | 4 |
| 14 | 1 | 0 | 4 |
| 15 | 1 | 0 | 4 |
| 16 | 1 | 0 | 4,5 |
| 17 | 1 | 0 | 4,5 |
| 18 | 1 | 0 | 5 |
| 19 | 1 | 0 | 5 |
| 20 | 1 | 0 | 5 |
| 21 | 1 | 0 | 5,5 |
| 22 | 1 | 0 | 5,5 |
| 23 | 1 | 0 | 5,5 |
| 24 | 1 | 0 | 6 |
| 25 | 1 | 0 | 6 |
| 26 | 1 | 0 | 6 |
| 27 | 2 | 3 | 2 |
| 28 | 2 | 3 | 2 |
| 29 | 2 | 3 | 2 |
| 30 | 2 | 3 | 2,5 |
| 31 | 2 | 3 | 2,5 |
| 32 | 2 | 3 | 2,5 |
| 33 | 2 | 0 | 3 |
| 34 | 2 | 0 | 3 |
| 35 | 2 | 0 | 3 |
| 36 | 2 | 0 | 3,5 |
| 37 | 2 | 0 | 3,5 |
| 38 | 2 | 3 | 3,5 |
| 39 | 2 | 0 | 4 |
| 40 | 2 | 0 | 4 |
| 41 | 2 | 0 | 4,5 |
| 42 | 2 | 0 | 4,5 |
| 43 | 2 | 0 | 4,5 |
| 44 | 2 | 0 | 5 |
| 45 | 2 | 0 | 5 |
| 46 | 2 | 0 | 5,5 |
| 47 | 2 | 0 | 5,5 |
| 48 | 2 | 0 | 6 |
| 49 | 2 | 0 | 6 |
| 50 | 2 | 0 | 6 |
| 51 | 3 | 3 | 2 |
| 52 | 3 | 3 | 2 |
| 53 | 3 | 3 | 2 |
| 54 | 3 | 3 | 2,5 |
| 55 | 3 | 3 | 2,5 |
| 56 | 3 | 3 | 2,5 |
| 57 | 3 | 3 | 3 |
| 58 | 3 | 3 | 3 |
| 59 | 3 | 3 | 3 |
| 60 | 3 | 3 | 3,5 |
| 61 | 3 | 3 | 3,5 |
| 62 | 3 | 3 | 3,5 |
| 63 | 3 | 0 | 4 |
| 64 | 3 | 3 | 4 |
| 65 | 3 | 3 | 4 |
| 66 | 3 | 3 | 4,5 |
| 67 | 3 | 0 | 4,5 |
| 68 | 3 | 0 | 4,5 |
| 69 | 3 | 0 | 5 |
| 70 | 3 | 0 | 5 |
| 71 | 3 | 0 | 5 |
| 72 | 3 | 0 | 5,5 |
| 73 | 3 | 0 | 5,5 |
| 74 | 3 | 0 | 5,5 |
| 75 | 3 | 3 | 6 |
| 76 | 3 | 0 | 6 |
| 77 | 3 | 0 | 6 |
| 78 | 3 | 0 | 6 |

**Supplementary data file S3, Final model**

$PROB FINAL MODEL TREATMENT OUTCOME MOUSE TB MODEL

$INPUT ID COMB DV TIME EVID

; COMB = drug combination (1 = RpZHE, 2 = RZMH)

; DV = dependent variable (0 = cure, 3 = failure)

; TIME = treatment time in months

$DATA ... IGNORE=@

$PRED

BASE = THETA(1)*EXP(ETA(1))

EMAX = THETA(2)

GAM = THETA(4)

IF(COMB.EQ.1) T50 = THETA(3)

IF(COMB.EQ.2) T50 = THETA(5)

PROB = BASE*(1-EMAX*TIME**GAM/(T50**GAM+TIME**GAM))

; for simulation

IF(ICALL.EQ.4) THEN

CALL RANDOM (2,R)

DV=2

RDV=1

IF(R.GT.PROB) DV=0

IF(R.GT.PROB) RDV=0

ENDIF

IF(DV.GT.1) THEN

Y=PROB

RDV = 1

ENDIF

IF(DV.LE.1) THEN

Y=1-PROB

RDV = 0

ENDIF

$THETA 1 FIX ; 1 BASE

$THETA 1 FIX ; 2 EMAX

$THETA (0,2.86) ; 3 T50_(RpZHE)

$THETA (0,9.14) ; 4 GAMMA

$THETA (0,4.35) ; 5 T50_(RZMH)

$OMEGA 0 FIX ; 1

;Sim_start (for VPC)

;$SIM (12345) (678910 UNI) ONLY NOP NSUB=100

$ESTIM MAXEVAL=9999 METHOD=COND LIKE PRINT=1 MSFO=msfb

$COV PRINT=E

;Sim_end for VPC

$TABLE ID DV PRED TIME RDV NOPRINT ONEHEADER FILE=sdtab

$TABLE ID COMB TIME NOPRINT ONEHEADER FILE=cotab

$TABLE ID NOPRINT ONEHEADER FILE=catab

$TABLE ID NOPRINT ONEHEADER FILE=patab
